# Multiplexed Single-Molecule Detection of Nucleic Acid Biomarkers with a Distance-Tuned Single FRET Pair

**DOI:** 10.1021/acs.analchem.5c01039

**Published:** 2025-08-23

**Authors:** Srishty Sethi, Kalani M. Wijesinghe, Md Monirul Islam, J. Chuck Harrell, Soma Dhakal

**Affiliations:** † Department of Chemistry, 6889 Virginia Commonwealth University, Richmond, Virginia 23284, United States; ‡ Department of Pathology, School of Medicine, 6889 Virginia Commonwealth University, Richmond, Virginia 23298, United States; § Massey Comprehensive Cancer Center, 6889 Virginia Commonwealth University, Richmond, Virginia 23298, United States

## Abstract

Biomarkers have gained tremendous attention in recent years, as they offer reliable detection of diseases such as cancers and other health conditions. However, with the recent realization that one biomarker can be associated with more than one disease (cross-talk), there is a significant shift toward simultaneous monitoring of more than one biomarker to increase the accuracy of diagnosis. Despite a sizable effort made over the last several years, multiplexing using the common techniques including surface-enhanced Raman spectroscopy (SERS), microarrays, RT-qPCR, nanostring, fluorescence, and others requires target amplification, target labeling, or the use of additional probes/actuators, and hence, these requirements complicate the experiments and data analyses. Using single-molecule fluorescence resonance energy transfer (smFRET) and 4-way DNA junction strategy, we introduced a relatively simple platform for simultaneous detection of multiple nucleic acid biomarkers. While the traditional way of smFRET-multiplexing requires multiple excitation sources and multiple FRET pairs (donor–acceptor dyes), our approach requires only one set of excitation sources and one FRET pair. Specifically, we presented four sensors that provide nonoverlapping FRET traces upon target binding, thus offering a clear multiplexed detection. Further, the 4-way DNA strategy enables detection down to low femtomolar (×10^–15^ M) level without requiring target labeling and amplification. Therefore, this simple and sensitive detection platform can benefit clinical diagnosis by offering early detection of diseases, including cancers.

## Graphical abstract

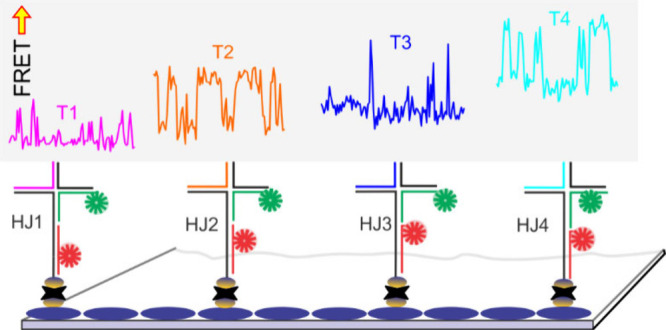

## Introduction

In recent years, nucleic acid biomarkers have been found to be increasingly important for early detection of many infectious diseases, neurodegenerative diseases, genetic abnormalities, and cancers.^1–3^ Their remarkable stability in body fluids makes them important candidates for screening various types of cancers that can substitute current painful invasive biopsies.^4–6^ Therefore, various ingenious technologies have been developed for the detection of nucleic acid biomarkers, which has captured significant attention in clinical diagnosis and monitoring disease progression, especially cancers.^5,7–9^ Despite noteworthy efforts, the World Health Organization reports that cancer is still the second leading cause of death in 112 countries. Strikingly, the 2020 GLOBOCAN database has projected a 47% increase in cancer-related deaths from 2020 to 2040.^10,11^ The primary reason for the high cancer mortality today is the lack of accurate and early detection, even though a timely diagnosis can offer more treatment options and increased survival rates. Since most nucleic acid-based detection techniques identify a single biomarker, which can be associated with multiple diseases,^12–14^ making early cancer detection challenging. Therefore, simultaneous monitoring of multiple biomarkers (multiplexing) is highly desirable to improve the accuracy of early diagnosis.^15^

Multiplexing offers several advantages over single-plex detection, as it can minimize false positives by reducing cross-talk and saves cost and time as compared to multiple single tests. Multiplexed detection also provides more reliable detection.^15^ Several laboratories have reported methods for multiplexed detection of nucleic-acid biomarkers, but they are not free from limitations. For example, a combination of lateral recombinase polymerase amplification (RPA) with a lateral flow device is fast and field-amenable but requires target amplification and labeling.^15^ Multiplexing using advanced gene editing-based technology SHERLOCK with CRISPR enzymology is an ultrasensitive technique but requires isothermal preamplification of target genetic material and complicated predesigning.^16^ RT-qPCR^12,17^ and surface-enhanced Raman spectroscopy (SERS)^18,19^ can detect nucleic acids in clinical samples, but they often require labeling of targets and/or enzymatic amplification, which can introduce false positives. While there are many more available multiplexed detection techniques, it is worth mentioning that DNA barcodes, microarray, and bulk fluorescence are capable of high throughput analysis; however, they require large sample size and have low sensitivity when not amplified^20–22^ (Table S1). Although various ensemble methods are emerging for multiplexed detection, if designed properly, the single-molecule methods can offer such capability without missing the unique properties of individual molecules^23^ (Figure S1). For example, single molecule techniques can capture conformational dynamics within the molecules that can be invaluable for sensor design and optimization. With careful designing, these techniques can be sensitive to identify fully complementary targets from the ones with single-nucleotide mismatches.^24,25^

In this regard, single-molecule fluorescence resonance energy transfer (FRET) has been leveraged for multiplexed detection.^26–28^ Specifically, single-molecule FRET is one of the few techniques that can have as low as millisecond time resolution and subnanometer temporal resolution, which is often necessary to resolve binding interactions and conformational dynamics of biomolecules.^29–31^ However, multiplexing using single-molecule FRET is still challenging as it requires either multiple excitation sources^26,27^ and/or multiple FRET pairs, which complicates not only the experiment but also the data analysis. Multiplexing using single-molecule FRET along with strand-displacement strategies have been reported before,^32,33^ but they are not ideal as the typical sensitivity would be at the picomolar level. Given the ultralow concentration in the biological samples (low pico- to femto-molar level^12,34–36^ ), it makes the detection of nucleic acid biomarkers quite challenging. Increasing the length of the binding region (number of base-pairs) between the probe and target sequence can provide higher binding stability and sensitivity, but it will compromise specificity. Considering these challenges, our group previously reported a FRET-based 4-way junction (HJ) strategy for the detection of nucleic acid biomarkers with low femtomolar sensitivity and high specificity.^25,37^ In this manuscript, we leveraged the dynamic nature of the 4-way junctions^38,39^ into multiplexed detection while still using a single FRET pair (Figure 1).

**Figure 1:**
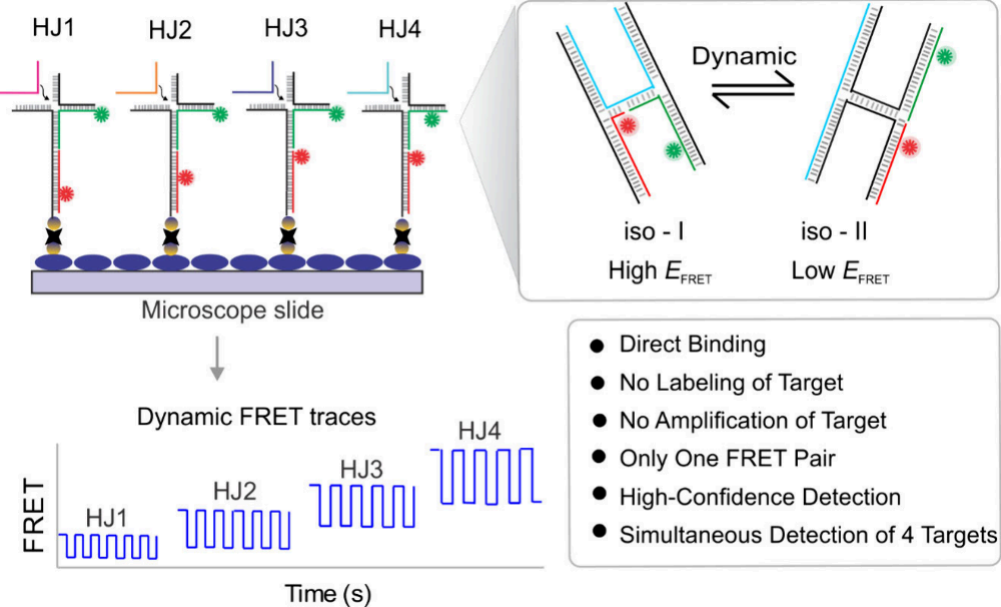
Single-molecule multiplexing using FRET-based detection. The DNA constructs form the four-way structure called Holliday junctions (HJ1, HJ2, HJ3, and HJ4) in the presence of targets. The targets can be distinguished based on the unique two-state FRET fluctuations between the iso-I and iso-II resulting from the inherent dynamics of the junction.

Herein, we designed four different four-way junctions (HJ1, HJ2, HJ3, and HJ4) with clear nonoverlapping FRET efficiency suitable for multiplex detection, which we first verified by testing the individual probe-target pair and then used all 4 targets simultaneously. In this proof-of-concept multiplexed platform, we utilized DNA mimics of miRNA biomarkers associated with triple-negative breast cancers (TNBCs)^40–42^ and showed that the platform is suitable for both single-plex and multiplexed detection with low femtomolar (fM) sensitivity. The produced FRET traces are unique identifying targets. Further, the kinetic analysis of the single-molecule traces shows that all four junctions produced distinct kinetic signatures, providing another level of scrutiny for target assignments in multiplexed experiments. Despite single-molecule fluorescence resonance energy transfer (smFRET) experiments being somewhat involved and requiring careful handling of the TIRF microscope, the advantages outweigh the technical challenges. In summary, this proof-of-concept multiplexed platform using the 4-way junction strategy offers several advantages including simplicity of design, direct binding without target modifications, no need of signal amplification, and secondary probes to achieve low femtomolar sensitivity and is free from false positives. Another advantage of this approach is that this platform can be easily customized to detect any sequences by redesigning the 4-way junctions and has high promise for early detection of cancers like TNBC and other diseases via specific and multiplexed detection of nucleic acid biomarkers.

## Results and Discussion

### Assembly and Selection of 4-Way Junctions (HJs)

To prevail the single-molecule FRET technique in multiplexing without requiring multiple excitation sources, complex labeling schemes, and sophisticated data analysis, we rationally designed and tested several DNA constructs (probes) with systematic FRET (donor–acceptor) labeling schemes. The goal was to identify the labeling schemes that provide nonoverlapping FRET efficiencies so that they can be used simultaneously without losing the ability to identify all targets. For this, we explored several designs (Figure S2) before settling into the four that provide sufficiently distinct FRET level and dynamics. To get the desired DNA constructs, each junction was assembled separately by thermal annealing of constituent DNA strands, one of which is biotinylated to allow surface immobilization on a quartz slide via biotin–streptavidin interactions; two strands are fluorophore-modified (Cy3 donor and Cy5 acceptor) to enable FRET, and two strands are complementary to the target sequence.^25^ The buffer used was 1× TAE (40 mM Tris, 20 mM acetic acid, and 1 mM EDTA) with 100 mM Mg^2+^. The target-binding region is the two ∼11 nucleotide arms of the junction, which was designed to be fully complementary to the target sequence. As complementary evidence, the successful annealing of the 4-way construct was validated previously using native polyacrylamide gel electrophoresis (PAGE).^25^ Further, these junctions were intact for days when stored at 4 °C providing a great level of flexibility for planning experiments.

Regarding the fluorophore labeling schemes, to achieve nonoverlapping FRET states, we positioned Cy3 and Cy5 fluorophores at various internal and terminal positions of the junction. The output FRET efficiencies were then determined via single-molecule FRET experiments. The rationale is that the junction with different labeling schemes and unique sequence at the binding region will enable the binding of respective targets and completes the 4-way structure, which then results in continuous switching into two distinct low- and high-FRET isomers (iso-I and iso-II). Out of the 13 designs tested (Figures S2 and S3), we selected four junctions that showed clear nonoverlapping FRET states. The same four junctions were then used for multiplexing. The complete list of DNA sequences used for the selected 4 junctions is shown in Table S2.

### Single-plex Detection

To ensure that the individual sensor constructs functioned as expected, we tested them using their respective target sequences. For this purpose, we selected DNA mimics of miR-107, miR-342-3p, miR-18b-5p, and miR-92a-3p (Table 1), which are known to be upregulated miRNAs during TNBC.^41,43,44^ The design was such that the annealed constructs (Figure 2) would have complementary regions for the target on strands B and D (half and half). In these experiments, the construct was first immobilized on the microscope slide and then the target sequence was injected and incubated for about 20 min before imaging. The single-molecule FRET experiments were performed using a prism-based total internal reflection fluorescence (pTIRF) microscope as described.^37,38,46^ Briefly, each construct type was separately immobilized on the microscope slide and washed by injecting the imaging buffer (1× TAE with 100 mM Mg^2+^) to remove any excess/unbound molecules. Note that the imaging buffer was supplemented with an oxygen scavenger system (OSS) to prevent photoblinking and to delay photobleaching upon illumination.^47^ In the pTIRF microscope, the incident green laser (532 nm) was used to excite the donor fluorophore (Cy3) and fluorescence emissions for Cy3 and Cy5 were recorded on an EMCCD camera at 10 frames per second. The red laser (639 nm) was illuminated at around 1000 frames (∼halfway via data acquisition) to confirm the presence of the Cy5 fluorophore. The fluorescence movies were then processed to first extract fluorescence-time traces using the IDL program and analyzed in MATLAB. The FRET efficiency (*E*_FRET_) was calculated as a ratio of the acceptor intensity (*I*_A_) to the sum of both the acceptor (*I*_A_) and donor intensities (*I*_D_). Finally, molecules showing proof of the presence of both donor and acceptor fluorophores were selected for further analysis.

**Table 1:** List of Targets Used for Individual Detection and for Multiplexing

S.N.	Targets	HJ Construct	Target Sequence
1	DNA mimic of miR-107	HJ1	AGC AGC ATT GTA CAG GGC TAT CA
2	DNA mimic of miR-342-3p	HJ2	TCT CAC ACA GAA ATC GCA CCC GT
3	DNA mimic of miR-18b-5p	HJ3	TAA GGT GCA TCT AGT GCA GTT AG
4	DNA mimic of miR-92a-3p	HJ4	TAT TGC ACT TGT CCC GGC CTG T

**Figure 2:**
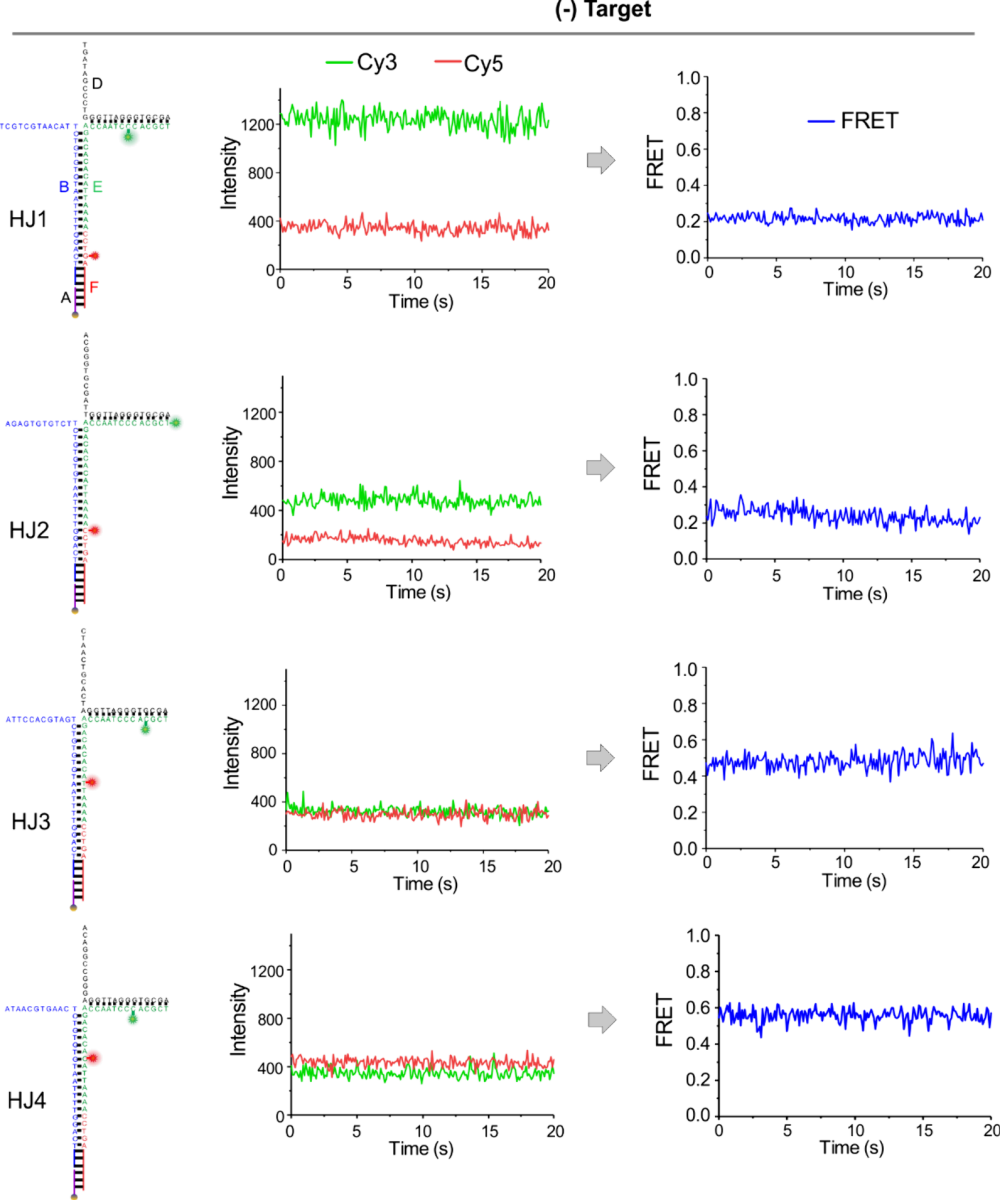
Typical intensity–time and corresponding FRET efficiency traces for the 4 junctions in the absence of the target. The junctions HJ1, HJ2, HJ3, and HJ4 were designed to detect miR-107, miR-342-3p, miR-18b-5p, and miR-92a-3p, respectively. In the absence of targets, the constructs show relatively static FRET traces. All of the experiments were carried out at room temperature.

Around 100 single molecules were selected to confirm the FRET behavior of the individual constructs. Figure 2 shows typical intensity–time traces and their corresponding FRET–time traces in the absence of targets. Interestingly, the fluorescence intensity for each of the incomplete junctions is relatively static FRET values of ∼0.2, ∼0.3, ∼0.4, and ∼0.6 for HJ1, HJ2, HJ3, and HJ4, respectively.

After confirming the successful formation of sensor constructs via single-molecule experiments, we tested them separately for the detection of respective targets. For this, we injected a desired concentration of target in the flow cell containing surface-immobilized constructs and then recorded movies after about 20 min of incubation. It is expected that, after the addition of the target, the incomplete junction would convert to a complete junction resulting in continuous conformational switching (Figure 1). As expected, an anticorrelated switching of fluorescence intensity of the Cy3 and Cy5 dynamic FRET traces was observed (Figure 3). More example FRET traces are included in the supporting text (Figures S4, S5, S6, and S7). Not only did the switching between low and high *E*_FRET_ states confirm the detection of target, but also the different FRET levels for different HJs unequivocally identified the targets. For example, continuous switching of two FRET states is as follows: HJ1 ∼ 0.2 ↔ 0.4, HJ2 ∼ 0.2 ↔ 0.6, HJ3 ∼ 0.3 ↔ 0.7, and HJ4 ∼ 0.4 ↔ 0.9. It is important to note that the continuous switching is inherent behavior of 4-way junctions, which not only makes the experiment interesting but also enables a high-confidence detection due to the overwhelmingly clear signal (continuous FRET switching) as compared to a one-step increase or decrease in FRET in most traditional FRET assays.

**Figure 3:**
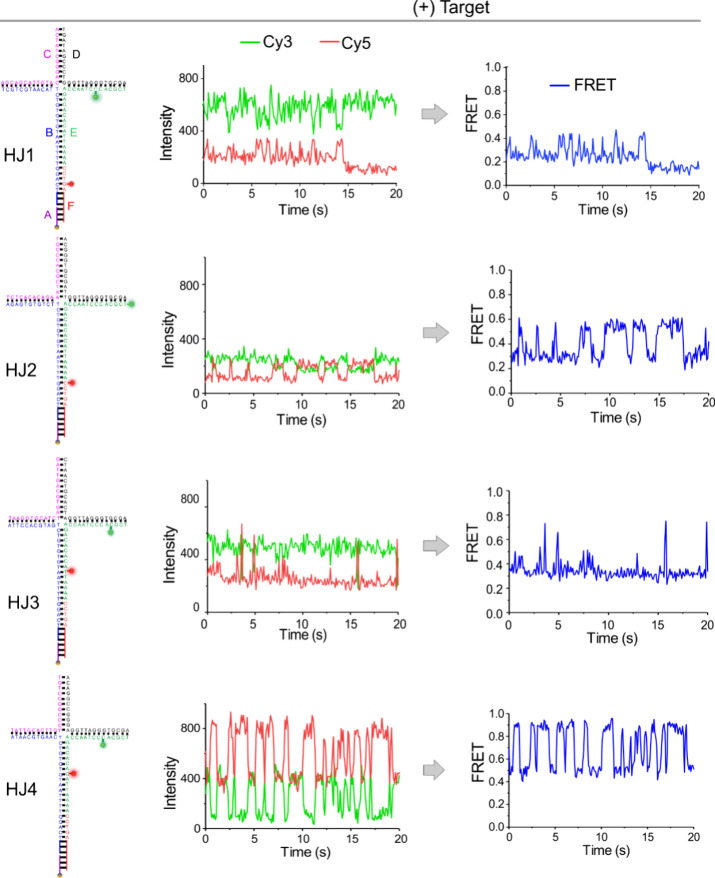
Typical intensity–time and corresponding FRET efficiency traces for the 4 junctions in the presence of target. The presence of corresponding targets (DNA mimics of miR-107, miR-342–3p, miR-18b-5p and miR-92a-3p) completes the formation of junction leading to anticorrelated dynamics of Cy3 (green) and Cy5 (red) intensities. The continuous switching between the two FRET states (HJ1 = ∼0.2 ↔ 0.4, HJ2 = ∼0.2 ↔ 0.6, HJ3 = ∼0.3 ↔ 0.7, and HJ4 = ∼0.4 ↔ 0.9) demonstrates the successful detection of targets. Experiments were carried out at room temperature, and traces shown were collected at 1 μM target concentration.

We also performed a kinetic analysis of the dynamic FRET traces using a well-established algorithm called hidden Markov model (HMM)^48–50^ and transition density plots (TDPs). The HMM analysis is used to determine the time-binned FRET transition states and their interconversion rates from dynamic single-molecule FRET traces. This algorithm utilizes the probability distribution of various FRET states and quantifies dwell times (underlying time sequence) for each FRET state.^48^ To determine the switching kinetics of each of the 4 junctions (HJ1, HJ2, HJ3, and HJ4), FRET traces for ∼200 dynamic molecules at a specific time frame (before photobleaching of fluorophore) were stitched into one trace and fit globally using HMM as shown in Figure 4. The TDP plots resulting from the two-state HMM analysis capture the most frequently occurring FRET states.

**Figure 4:**
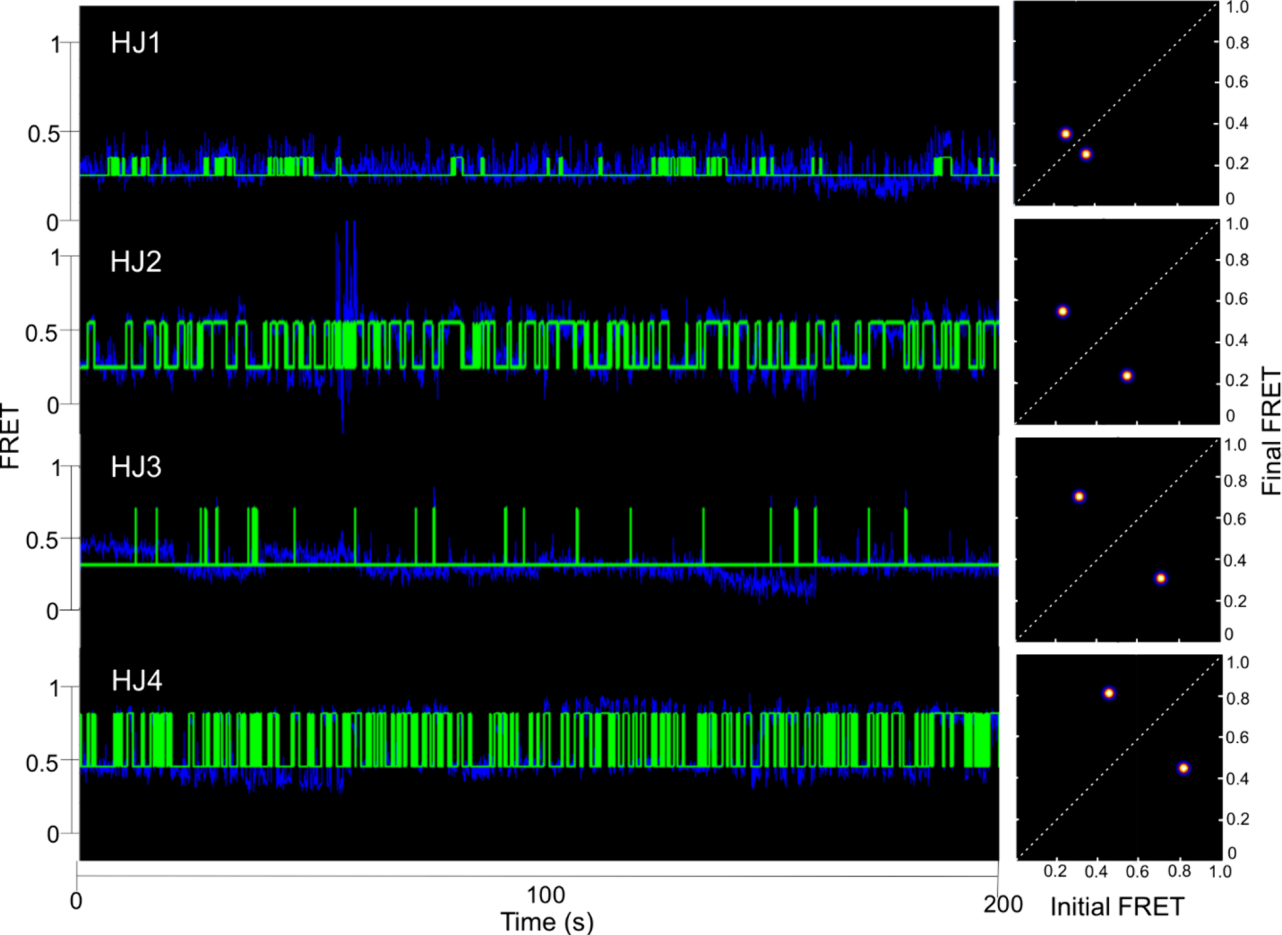
HMM and TDP analysis of the dynamic traces for HJ1, HJ2, HJ3, and HJ4. Left: HMM fitting of dynamic FRET traces. The FRET traces are shown in blue, and the HMM fittings are shown in green. A small portion (10 molecules) of the globally fitted dynamic single-molecules is shown. Right: Transition density plots (TDPs). Each bright spot represents the most frequent transition within a population of molecules as a heat-map. Two transitions were obtained for each junction with a distinguishable bright spot. The total number of dynamic molecules used for HMM/TDP analysis was 213, 165, 213, and 325 for HJ1, HJ2, HJ3, and HJ4, respectively.

Briefly, the HMM analyses were performed on dynamic traces from each 4-way junction. The analysis resulted in clear low- and high-FRET states that are consistent with the visually clear two FRET states presented in the typical dynamic traces obtained from the smFRET experiments (Figure 3). Further, the TDP analysis, which extracts quantitative kinetic information on conformational dynamics from the HMM fitted data, showed a reversible conformational change with a line of symmetry (Figure 4).^46,48^ We procured the rate of the low- to high-FRET (*k*_2→1_) and high- to low-FRET (*k*_1→2_) transitions for each HJ from TDPs (Table 2) and then the equilibrium constant (*K*_eq_) using the equation below.

$$\mathrm{Equilibrium}\;\mathrm{constant},K_{eq}=\frac{k_{2\to 1}}{k_{1\to 2}}$$

**Table 2:** Comparison of HMM/TDP Extracted Transition Rates of HJ1, HJ2, HJ3, and HJ4

Designs	Transitions	Mean Rate (*k*) ± SD (s^–1^)	#Transitions (*N*)	$K_{\mathrm{eq}}=\frac{k_{2\to 1}}{k_{1\to 2}}$
HJ1-107^a^	2 → 1	0.25 ± 0.06	672	4 ± 1^a^
	1 → 2	0.06 ± 0.02	672	
HJ2-342-3p	2 → 1	0.7 ± 0.2	1409	0.8 ± 0.3
	1 → 2	0.8 ± 0.2	1409	
HJ3-18b-5p	2 → 1	6 ± 2	428	50 ± 20
	1 → 2	0.11 ± 0.03	428	
HJ4-92a-3p	2 → 1	1.5 ± 0.4	3657	1.2 ± 0.4
	1 → 2	1.2 ± 0.3	3656	

a Due to fast transitions (small dwell time at the high-FRET level), the HMM fitting missed some of the transitions for HJ1-107.

While similar rates for 2 → 1 and 1 → 2 will result in *K*_eq_ ≅ 1 representing similar dwell times for the low and high FRET state, what is observed is *K*_eq_ ≠ 1 (4, 0.8, 50, and 1.2 for HJ1, HJ2, HJ3, and HJ4, respectively), which means that these HJs assume a shorter dwell time in one of the states than the other and that they are distinctly different (Table 2). In fact, these different *K*_eq_ values can help identify targets. It is important to mention that these results are consistent with the length of dwell times and frequency of transitions, as expected from raw FRET traces. Therefore, in addition to the FRET values that a particular HJ spans, they also yield a different FRET pattern, which provides a second level of assurance in identifying targets. In addition, the level of initial and final FRET for each of the junctions in TDPs is visually different from one another (position and distance between the bright spots), demonstrating that TDPs can also be used as complementary evidence in retroactively identifying these junctions/targets.

### Analytical Sensitivity and Limit of Detection

Next, to determine the analytical sensitivity for each of the targets, we performed FRET measurements for a series of target concentrations. In these experiments, we counted the number of dynamic molecules (target-bound) as a function of the concentration. The approach used here is essentially digital counting that enables detection with high confidence. Specifically, we determined the percentage of dynamic molecules by dividing the number of molecules showing clear dynamics by the total number of single molecules, as shown in the equation below.

$$\%\;Dynamic\;molecules=\frac{Number\;\mathrm{of}\;dynamic\;molecules}{Total\;number\;\mathrm{of}\;\sin gle\;molecules}\times 100\%$$

When the % dynamic molecules were plotted against the concentration of target for each of the HJ, it is clear that the population of dynamic molecules increased up to a certain concentration of target and then plateaued (Figure 5). A linear range of the curves is shown in Figure S8. Since the experiments performed in the absence of targets (negative control) did not yield such dynamic molecules, the limit of detection (LOD) in these experiments is defined to be the lowest concentration of the target at which dynamic molecules are still found.

**Figure 5:**
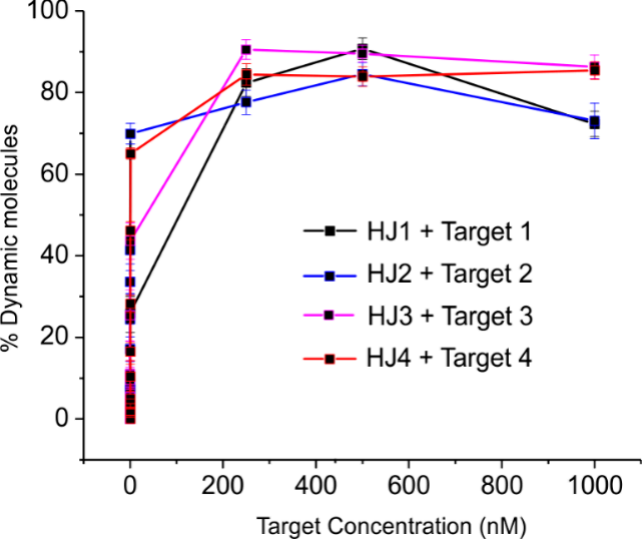
Determination of the sensor response as a function of target concentration. The curve displays % dynamic molecules as a function of target concentration for all 4 HJs. Targets used (Targets 1–4) were DNA mimics of miR-107, miR-342-3p, miR-18b-5p, and miR-92a-3p, respectively. About 100 single molecules were selected at each concentration and randomly assigned to 3 groups to determine the mean % dynamic molecules and standard deviation.

The concentration dependent analysis shows that the HJ1, HJ2, HJ3, and HJ4 can detect targets down to the 10–50 fM range, which corresponds to ∼1–5 attomoles considering the 100 μL sample size we used (Figure 5). We observed ∼0.8% and ∼4.3% dynamic traces at 50 fM targets for HJ1 and HJ2 and ∼1.2% and ∼1.8% dynamic traces at 10 fM for HJ3 and HJ4, respectively. Below these concentrations, no dynamic molecules were observed, defining the limit of detection (LOD) for these targets. Further, the curves show that the dynamic range for the HJ-based sensor is up to ∼100–200 nM. Nevertheless, the large dynamic range and the attomole level detection without requiring target amplification and labeling make this platform quite promising for clinical detection of nucleic acid biomarkers.

### Multiplexing

The HJ-based detection platform was also tested for detection of 4 targets simultaneously. The goal was to test whether the presented single-molecule FRET technique is suitable for multiplexing in a simple platform that does not require multiple FRET pairs or multiple excitation sources. Motivated by this goal, for a proof-of-concept test, we attempted the detection of 4 different targets on the same slide simultaneously by injecting a premix of 4 HJs followed by a premix of 4 targets. For this, the 4 HJs with different interdye distances as characterized above were separately thermal annealed and premixed in an equimolar ratio and immobilized onto the microscope slide. After washing the unbound HJs with the imaging buffer, an equimolar ratio of all 4 targets (DNA mimics of miR-107, miR-342-3p, miR-18b-5p, and miR-92a-3p) was mixed and injected into the flow cell. Movies were recorded after a 20 min incubation period. An experimental setup of the microscope slide and the intensity–time traces as well as corresponding FRET traces are shown in Figure 6A. When the traces were analyzed, out of the total 498 single molecules collected, 434 molecules showed dynamic switching. These traces were easily classified into 4 types based on their intensity–time as well as FRET profiles (Type 1 to Type 4, Figure 6A), which were then assigned to specific HJs based on the expected FRET outcomes of each HJ in Figure 3. The analysis showed that the percentages of Type 1, Type 2, Type 3, and Type 4 molecules were 26.4%, 13.2%, 18.2%, and 31.8%, corresponding to HJ1, HJ2, HJ3, and HJ4, respectively. Although equal amounts of HJs followed by equal amounts of targets were used, the small difference in the percentage of dynamic molecules observed may be attributed to factors such as the difference in sequences of targets and their possible secondary structures. Nonetheless, this experiment demonstrated a successful detection of 4 targets simultaneously using the HJ-based strategy. Furthermore, this strategy may be scaled up to ∼5-fold multiplexing with the help of the MASH-FRET program that assists in the identification of FRET traces even with smaller differences in FRET values.^51^

**Figure 6:**
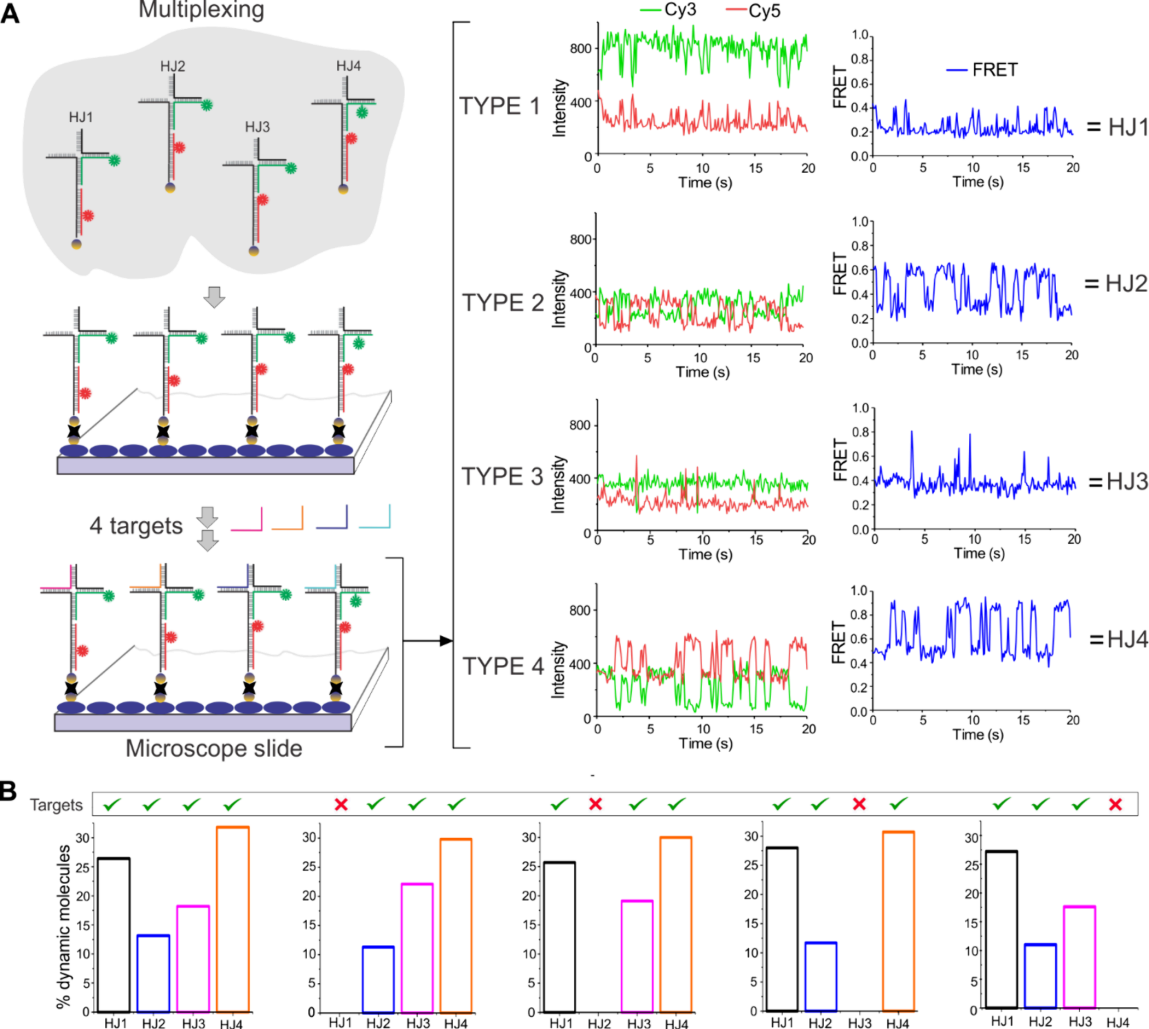
Multiplexing and specificity test. (A) Multiplex detection. Left: Experimental setup of simultaneous immobilization of four HJ constructs followed by injection of the premixed 4 targets on the same microscope slide (1 μM each). Middle and Right: Four typical types of intensity–time (TYPE 1, TYPE 2, TYPE 3, and TYPE 4) and hence the FRET traces were observed, identifying HJs and their corresponding targets. (B) Specificity test. The binding of the target was determined for the four sensors (HJ1, HJ2, HJ3, and HJ4) for two cases: (i) when all four targets were present (left plot) and (ii) when one of the targets was missing (identified by a cross mark). The targets were kept at 1 μM, and no cross-binding was observed even at this saturating concentration. The total number of single molecules analyzed was 498, 389, 413, 411, and 426 respectively, from the left to the right plot.

Further, to determine the specificity in the multiplexed detection, we performed a series of tests where one of the four targets was missing at a time, while all four sensors were simultaneously present on the microscope slide (Figure 6B). It is important to note that, in these systematic omission experiments, the three target sequences act as nonspecific targets for the HJ whose own target is missing. Unlike in the 4-target experiments where 4 types of dynamic FRET traces were observed (positive control), only three types of traces corresponding to their respective sensor constructs were obtained in each omission test. For example, no evidence of HJ1 type FRET dynamics was observed in the absence of target 1 (DNA mimic of miR-107). These results demonstrated the high specificity of HJ-based detection even at the saturating concentration (1 μM) of nonspecific targets. In addition, our group previously demonstrated that the HJ-based strategy with ∼11bp arms (similar to ones used here) can discriminate the target with single-nucleotide mismatches when present at the core of the junction or in the middle of the arms,^25^ offering a high level of specificity.

Overall, the single-molecule detection platform demonstrated here has several advantages. From a technology point of view, the use of only one FRET pair and only one pair of excitation sources simplifies the technology. This is because the need for multiple FRET pairs and excitation sources is one of the obstacles in extending single-molecule FRET in multiplexing. Unlike typical toehold-based strand development approaches that are developed for nucleic acid detection, it is noteworthy to mention that the developed HJ-based approach enables direct binding and detection at the low fM level without target labeling and amplification. Therefore, the multiplexing of which as demonstrated here can provide high confidence detection of diseases, including cancers.

## Material and Methods

### DNA Sequences and Chemical Reagents

All the DNA sequences including biotinylated DNA and fluorophore modified strands were acquired in an amorphous powder from Integrated DNA Technologies (IDT) Inc., dissolved in filtered sterile water to a final concentration of 100 μM, and stored at −20 °C until needed. Biotinylated bovine serum albumin (bBSA), magnesium chloride hexahydrate, potassium chloride, tris­(hydroxymethyl)-aminomethane (Tris), acetic acid, ethylenediaminetetraacetic acid disodium salt (EDTA), 6-hydroxy-2,5,7,8-tetramethylchroman-2-carboxylic acid (Trolox), and protocatechuate 3,4-dioxygenase (PCD), were purchased from Fisher Scientific. Streptavidin and protocatechuic acid (PCA) were purchased from VWR.

### Assembly of 4-Way Junctions

For the assembly of 4-way junctions, we thermally annealed five single-stranded DNA oligonucleotides (Table S2) at 1 μM concentration. This is done separately for each junction based on the target and the fluorophore positions. An equimolar (1 μM) ratio of oligonucleotides for each junction design was mixed in a buffer containing 100 mM Mg^2+^ and 1× TAE (40 mM Tris, 20 mM acetic acid, and 1 mM EDTA) separately. Thermal annealing was carried out in a thermal cycler (BIO-RAD, T100), where the sample was heated to 95 °C for 5 min and then slowly cooled to 4 °C in the duration of ∼2 h as described previously.^46,52^ Once annealed, these samples were stable for several days when stored at 4 °C.

### Preparation of Flow Cell

In smFRET, precleaned quartz slides (75 × 26 × 1 mm) were used to make flow cells. For this, two holes were drilled diagonally, and an L-shaped channel was prepared using parafilm, a glass coverslip (24 × 60 mm, Fisher Scientific), pipet tips (200 μL plastic tips), and tubing (0.02 in. ID, 0.06 in. OD, Cole-Palmer), as reported in our previous publications. All of the connections were sealed using epoxy. For the smFRET experiments, the flow cell was first surface functionalized by consecutive injections of 1 mg/mL biotinylated BSA (incubate for 5 min) and 0.2 mg/mL streptavidin (incubate for 2 min), respectively. Finally, ∼300 μL of 1× TAE-Mg buffer was injected to remove the unbound molecules.^32,53–55^ We estimate nearly 100–150 μL of buffer is required to completely fill the flow cell.

### Single-Molecule Sample Preparation and Fluorescence Microscopy

The functionalized flow cell was placed on the microscope stage and the fluorescence background was photobleached by illuminating the flow cell with both the green (532 nm) and red (639 nm) lasers together.^53^ After photobleaching, the preannealed 4-way DNA junction (without target) was injected in the flow cell. Briefly, ∼5–30 pM solution of preannealed junction was prepared in a buffer of 1× TAE, 100 mM MgCl_2_, and the oxygen scavenging system (OSS)^47^ containing 2 mM Trolox, 5 mM PCA, and 50 nM PCD, called imaging buffer. However, for multiplexing, an equimolar ratio of four separately annealed junctions (HJ1:HJ2:HJ3:HJ4 = 1:1:1:1) was prepared in the imaging buffer where the concentration of the individual sensor was 4 pM. The flow cell was incubated for about 30 s to allow immobilization of the biotinylated DNA junction on the quartz slide, and then, the flow cell was flushed with an imaging buffer to remove the unbound molecules. Later, the target solution (prepared in the same imaging buffer) was added. Please note that only one target is added for the single-plex detection, and an equimolar ratio of four targets was added for the multiplex experiment. FRET movies were recorded after ∼20 min of incubation. While recording FRET movies, the Cy3 fluorophore was continuously excited using a green laser and the fluorescence intensities of both the Cy3 and Cy5 fluorophores were recorded using an EMCCD camera at 100 ms time resolution. The EMCCD camera splits the field of view into two channels (512 × 256 pixels each) and visualizes single molecules as bright spots on the images. Toward the end of the experiment, the red laser was illuminated to photobleach the Cy5 fluorophore in order to confirm the presence of an active FRET pair. All of the single-molecule FRET experiments were performed at room temperature (23 °C).

### Data Acquisition and Analysis

The fluorescence movies obtained with the EMCCD camera were processed using smFRET data acquisition and the processing protocol described in our previous publications.^38,54^ Briefly, data acquisition was carried out in Single.exe software available from the Ha Lab (https://github.com/Ha-SingleMoleculeLab/Data-Aquisition), which generates the .pma files for each FRET movie, and later, the IDL program was used to extract fluorescence–time traces. Further, the MATLAB script was utilized to screen the single molecule from each movie, which shows evidence of both Cy3 and Cy5 fluorophores. Around 100 single molecules were selected manually from each experiment and separated into static and dynamic molecules.^25,37^ This is done by looking for the anticorrelation between two fluorophores. The dynamic molecules showed a clear anticorrelation representing a continuous transition between two FRET states in intensity–time traces. The .dat files from MATLAB were saved for the selected single-molecules and input in the OriginLab software to obtain intensity versus time plots. We manually calculated FRET efficiency using the equation (FRETorEFRET=IA(IA+ID)) . The percentages of dynamic molecules were calculated and plotted against the target concentration to generate calibration curves. The limit of detection (LOD) for each junction/target was determined based on the lowest concentration of the target that still yields dynamic molecules. The standard deviations (SDs) for each concentration data point were determined by randomly grouping single molecules into three groups (including both static and dynamic) at each target concentration. For multiplexing, single molecules were manually divided into five categories, which include static molecules and dynamic molecules from targets T1, T2, T3, and T4 based on their expected FRET ranges as determined from the one-on-one experiments.

### Hidden Markov Model (HMM) and Transition Density Plots (TDPs)

The dynamic FRET trajectories and kinetics were determined using the HaMMy and Transition density plot (TDP) analyses available at https://sites.google.com/site/taekjiphalab/resources. The HMM analysis for the FRET transitions was performed on the dynamic traces. Briefly, HMM is based on the probability of the most frequent FRET distribution in different states, their transition rates, and the dwell time of their underlying states.^48^ About 200 dynamic single molecules from each HJ (HJ1, HJ2, HJ3, and HJ4) with clear anticorrelation of the donor and acceptor intensities were truncated for a 20 s (200 frames) window using a custom MATLAB script to determine their comprehensive transition rates. The HaMMy program was first initialized to 2 transition states, and each truncated file was loaded and analyzed for their overall FRET transitions. Further, to perform a kinetic analysis of transition states obtained from each set of molecules from individual HJs, we used the TDP program. This program identifies the FRET population in their transition states and provides a two-dimensional plot of final vs initial FRET in the form of a heat map based on the frequency of FRET transition, in this case between the low- to high-FRET (2 → 1) and high- to low-FRET (1 → 2). TDP uses Gaussian-fitted histograms to calculate kinetic rates (*k*_2→1_ and *k*_1→2_) and standard deviations corresponding to the globally fitted dynamic molecules for individual junctions. All of the dynamic single-molecules used in the HMM and TDP analyses were obtained from the smFRET experiments performed at 1 μM target concentration.

## Conclusion

The multiplex detection platform demonstrated here utilizes a 4-way junction-based strategy for a clear and simultaneous detection of four DNA targets in one sample. This multiplex detection method not only reduces the cost and time compared to the single-plex detection but also can increase the reliability of diagnosis. The key feature of this platform involves simultaneous detection of four unlabeled targets without amplification, which clearly advances the conventional FRET-based single-molecule approaches. This simple design also simplifies the data analysis. It would be ideal to achieve many-fold multiplexing from a single channel; as of now, it is quite challenging without sophisticated labeling and multiple excitation approaches. One possibility going forward would be the use of the HJ-based multiplexed strategy developed here along with a multichannel flow cell (microscope slide) enabling 4-fold × *N*-plex multiplexing where *N* is the number of channels. Given that the multiplexed platform can be easily advanced to obtain more than one channel per microscope slide, it has the potential to boost multiplexing. Further, the use of a simple hybridization-based strategy in multiplexing allows detection of any nucleic acid targets with custom-designed sensors, and thus, it has transformative potential in the field of diagnostics.

## Supplementary Material



## Notes

Comparison of multiplexing methods; Multiplexed Bulk FRET; Designs of 4-way junctions; Estimated and experimental FRET efficiencies; List of DNA sequences; Typical single-molecule traces for HJ1; Typical single-molecule traces for HJ2; Typical single-molecule traces for HJ3; Typical single-molecule traces for HJ4; Linear range of calibration curves; Supporting references (PDF)

## References

[1] Grasso M., Piscopo P., Confaloni A., Denti M. (2014). Circulating miRNAs
as Biomarkers for Neurodegenerative Disorders. Molecules.

[2] Niemz A., Ferguson T. M., Boyle D. S. (2011). Point-of-Care Nucleic
Acid Testing
for Infectious Diseases. Trends Biotechnol..

[3] Rahat B., Ali T., Sapehia D., Mahajan A., Kaur J. (2020). Circulating Cell-Free
Nucleic Acids as Epigenetic Biomarkers in Precision Medicine. Front. Genet..

[4] Schwarzenbach H., Hoon D. S. B., Pantel K. (2011). Cell-Free Nucleic Acids as Biomarkers
in Cancer Patients. Nat. Rev. Cancer.

[5] Szilágyi M., Pös O., Márton É., Buglyó G., Soltész B., Keserű J., Penyige A., Szemes T., Nagy B. (2020). Circulating
Cell-Free Nucleic Acids: Main Characteristics and Clinical
Application. Int. J. Mol. Sci..

[6] Heitzer E., Haque I. S., Roberts C. E. S., Speicher M. R. (2019). Current and Future
Perspectives of Liquid Biopsies in Genomics-Driven Oncology. Nat. Rev. Genet..

[7] Kumar R. R., Kumar A., Chuang C.-H., Shaikh M. O. (2023). Recent Advances
and Emerging Trends in Cancer Biomarker Detection Technologies. Ind. Eng. Chem. Res..

[8] Johnson P., Zhou Q., Dao D. Y., Lo Y. M. D. (2022). Circulating Biomarkers
in the Diagnosis and Management of Hepatocellular Carcinoma. Nat. Rev. Gastroenterol. Hepatol..

[9] Schwarzenbach H. (2013). Circulating
Nucleic Acids as Biomarkers in Breast Cancer. Breast Cancer Res..

[10] Sung H., Ferlay J., Siegel R. L., Laversanne M., Soerjomataram I., Jemal A., Bray F. (2021). Global Cancer Statistics
2020: GLOBOCAN Estimates of Incidence and Mortality Worldwide for
36 Cancers in 185 Countries. CA. Cancer J. Clin..

[11] Ahmad F. B., Anderson R. N. (2021). The Leading Causes
of Death in the US for 2020. JAMA.

[12] Jet T., Gines G., Rondelez Y., Taly V. (2021). Advances in Multiplexed
Techniques for the Detection and Quantification of microRNAs. Chem. Soc. Rev..

[13] Cho W. C. (2007). OncomiRs:
The Discovery and Progress of microRNAs in Cancers. Mol. Cancer.

[14] Wang J., Zhang K.-Y., Liu S.-M., Sen S. (2014). Tumor-Associated Circulating
MicroRNAs as Biomarkers of Cancer. Molecules.

[15] Li J., Pollak N. M., Macdonald J. (2019). Multiplex Detection of Nucleic Acids
Using Recombinase Polymerase Amplification and a Molecular Colorimetric
7-Segment Display. ACS Omega.

[16] Gootenberg J. S., Abudayyeh O. O., Kellner M. J., Joung J., Collins J. J., Zhang F. (2018). Multiplexed
and Portable Nucleic Acid Detection Platform with Cas13,
Cas12a, and Csm6. Science.

[17] Wang Y., Zhou J., Chen Y., Wang C., Wu E., Fu L., Xie C. (2017). Quantification
of Distinct Let-7 microRNA Family Members
by a Modified Stem-Loop RT-qPCR. Mol. Med. Rep..

[18] Wang H.-N., Crawford B. M., Fales A. M., Bowie M. L., Seewaldt V. L., Vo-Dinh T. (2016). Multiplexed Detection of MicroRNA Biomarkers Using
SERS-Based Inverse Molecular Sentinel (iMS) Nanoprobes. J. Phys. Chem. C.

[19] Zhou W., Tian Y.-F., Yin B.-C., Ye B.-C. (2017). Simultaneous Surface-Enhanced
Raman Spectroscopy Detection of Multiplexed MicroRNA Biomarkers. Anal. Chem..

[20] Koch C., Reilly-O’Donnell B., Gutierrez R., Lucarelli C., Ng F. S., Gorelik J., Ivanov A. P., Edel J. B. (2023). Nanopore Sequencing of DNA-Barcoded
Probes for Highly
Multiplexed Detection of microRNA, Proteins and Small Biomarkers. Nat. Nanotechnol..

[21] Epstein J. R., Biran I., Walt D. R. (2002). Fluorescence-Based
Nucleic Acid Detection
and Microarrays. Anal. Chim. Acta.

[22] Jie G., Zhao Y., Wang X., Ding C. (2017). Multiplexed Fluorescence
Detection of microRNAs Based on Novel Distinguishable Quantum Dot
Signal Probes by Cycle Amplification Strategy. Sens. Actuators B Chem..

[23] Akkilic N., Geschwindner S., Höök F. (2020). Single-Molecule
Biosensors: Recent
Advances and Applications. Biosens. Bioelectron..

[24] Gilboa T., Garden P. M., Cohen L. (2020). Single-Molecule
Analysis of Nucleic
Acid Biomarkers–A Review. Anal. Chim.
Acta.

[25] Megalathan A., Wijesinghe K. M., Dhakal S. (2021). Single-Molecule FRET-Based Dynamic
DNA Sensor. ACS Sens..

[26] Stein I. H., Steinhauer C., Tinnefeld P. (2011). Single-Molecule Four-Color FRET Visualizes
Energy-Transfer Paths on DNA Origami. J. Am.
Chem. Soc..

[27] Bunt G., Wouters F. S. (2017). FRET from Single to Multiplexed Signaling Events. Biophys. Rev..

[28] Qiu X., Hildebrandt N. (2015). Rapid and Multiplexed MicroRNA Diagnostic Assay Using
Quantum Dot-Based Förster Resonance Energy Transfer. ACS Nano.

[29] Lerner E., Cordes T., Ingargiola A., Alhadid Y., Chung S., Michalet X., Weiss S. (2018). Toward Dynamic
Structural Biology:
Two Decades of Single-Molecule Förster Resonance Energy Transfer. Science.

[30] LeBlanc S., Kulkarni P., Weninger K. (2018). Single Molecule FRET: A Powerful
Tool to Study Intrinsically Disordered Proteins. Biomolecules.

[31] Gomes G.-N., Gradinaru C. C. (2017). Insights into the Conformations and Dynamics of Intrinsically
Disordered Proteins Using Single-Molecule Fluorescence. Biochim. Biophys. Acta BBA - Proteins Proteomics.

[32] Kaur A., Sapkota K., Dhakal S. (2019). Multiplexed Nucleic Acid Sensing
with Single-Molecule FRET. ACS Sens..

[33] Kaur A., Dhakal S. (2020). Recent Applications of FRET-Based Multiplexed Techniques. TrAC Trends Anal. Chem..

[34] Kuo C.-W., Smith A. M. (2023). Digital and Absolute Assays for Low
Abundance Molecular
Biomarkers. Acc. Chem. Res..

[35] Williams Z., Ben-Dov I. Z., Elias R., Mihailovic A., Brown M., Rosenwaks Z., Tuschl T. (2013). Comprehensive Profiling
of Circulating microRNA via Small RNA Sequencing of cDNA Libraries
Reveals Biomarker Potential and Limitations. Proc. Natl. Acad. Sci. U. S. A..

[36] D’Agata R., Spoto G. (2019). Advanced Methods for
microRNA Biosensing: A Problem-Solving Perspective. Anal. Bioanal. Chem..

[37] Wijesinghe K. M., Kanak M. A., Harrell J. C., Dhakal S. (2022). Single-Molecule Sensor
for High-Confidence Detection of miRNA. ACS
Sens..

[38] Gibbs D. R., Dhakal S. (2018). Single-Molecule Imaging Reveals Conformational
Manipulation
of Holliday Junction DNA by the Junction Processing Protein RuvA. Biochemistry.

[39] Ortiz-Lombardía M., González A., Aymamí J., Azorín F., Coll M. (1999). Crystal Structure of
a DNA Holliday Junction. Nat. Struct. Biol..

[40] Sabit H., Cevik E., Tombuloglu H., Abdel-Ghany S., Tombuloglu G., Esteller M. (2021). Triple Negative Breast
Cancer in
the Era of miRNA. Crit. Rev. Oncol. Hematol..

[41] Kleivi
Sahlberg K., Bottai G., Naume B., Burwinkel B., Calin G. A., Borresen-Dale A.-L., Santarpia L. (2015). A Serum MicroRNA
Signature Predicts Tumor Relapse and Survival in Triple-Negative Breast
Cancer Patients. Clin. Cancer Res..

[42] Liu J., Zhang L., Zeng W., Zhang L., He N., Lu Z. (2023). High-Throughput
Quantitative Detection of Triple-Negative Breast
Cancer-Associated Expressed miRNAs by Rolling Circle Amplification
on Fluorescence-Encoded Microspheres. Chin.
Chem. Lett..

[43] Piña-Sánchez P., Valdez-Salazar H.-A., Ruiz-Tachiquín M.-E. (2020). Circulating microRNAs
and Their Role in the Immune Response in Triple-negative Breast Cancer
(Review). Oncol. Lett..

[44] Shin V. Y., Siu J. M., Cheuk I., Ng E. K. O., Kwong A. (2015). Circulating
Cell-Free miRNAs as Biomarker for Triple-Negative Breast Cancer. Br. J. Cancer.

[46] Mahmoud R., Dhakal S. (2022). Single-Molecule Analysis
of DNA Branch Migration under
Biomimetic Environments. J. Phys. Chem. B.

[47] Aitken C. E., Marshall R. A., Puglisi J. D. (2008). An Oxygen Scavenging System for Improvement
of Dye Stability in Single-Molecule Fluorescence Experiments. Biophys. J..

[48] McKinney S. A., Joo C., Ha T. (2006). Analysis of
Single-Molecule FRET Trajectories Using
Hidden Markov Modeling. Biophys. J..

[49] Pirchi M., Ziv G., Riven I., Cohen S. S., Zohar N., Barak Y., Haran G. (2011). Single-Molecule Fluorescence Spectroscopy Maps the Folding Landscape
of a Large Protein. Nat. Commun..

[50] Landes C. F., Rambhadran A., Taylor J. N., Salatan F., Jayaraman V. (2011). Structural
Landscape of Isolated Agonist-Binding Domains from Single AMPA Receptors. Nat. Chem. Biol..

[51] Kaur A., Ellison M., Dhakal S. (2021). MASH-FRET:
A Simplified Approach
for Single-Molecule Multiplexing Using FRET. Anal. Chem..

[52] Wijesinghe K. M., Sabbih G., Algama C. H., Syed R., Danquah M. K., Dhakal S. (2023). FRET-Based Single-Molecule Detection
of Pathogen Protein
IsdA Using Computationally Selected Aptamers. Anal. Chem..

[53] Gibbs D., Kaur A., Megalathan A., Sapkota K., Dhakal S. (2018). Build Your
Own Microscope: Step-By-Step Guide for Building a Prism-Based TIRF
Microscope. Methods Protoc..

[54] DeSalle, R. , Ed. DNA Barcoding: Methods and Protocols. In Methods in Molecular Biology; Springer US: New York, NY, 2024; Vol. 2744; 10.1007/978-1-0716-3581-0.

[55] Roy R., Hohng S., Ha T. (2008). A Practical Guide to Single-Molecule
FRET. Nat. Methods.

