# Supplementary material for: Multiplexed Single-Molecule Detection of Nucleic Acid Biomarkers with a Distance-Tuned Single FRET Pair
Source: Anal Chem. 2025 Aug 23;97(35):18918–27. doi: 10.1021/acs.analchem.5c01039 (PMC12403156; doi:10.1021/acs.analchem.5c01039)
Supplement: Supplementary file 1 [file ac5c01039_si_001.pdf]

Supplementary information

Multiplexed Single-Molecule Detection of Nucleic Acid Biomarkers with a Distance-Tuned Single FRET Pair

Srishty Sethi[1], Kalani M. Wijesinghe[1], Md Monirul Islam[1], J. Chuck Harrell[2,3], Soma Dhakal[1,3]*

[1]Department of Chemistry, Virginia Commonwealth University, Richmond, VA 23284, USA

[2]Department of Pathology, School of Medicine, Virginia Commonwealth University, Richmond, Virginia 23298, United States

[3]Massey Comprehensive Cancer, Virginia Commonwealth University, Richmond, Virginia 23298, United States

*Correspondence: sndhakal@vcu.edu

Table of Contents *Page*

1. Comparison of multiplexing detection methods S2
2. Multiplexed Bulk FRET of the selected 4-way junctions S3
3. Designs of 4-way junctions S4
4. Estimated and Experimental FRET efficiencies S5
5. List of DNA sequences S6
6. Typical single-molecule traces for HJ1 S7
7. Typical single-molecule traces for HJ2 S8
8. Typical single-molecule traces for HJ3 S9
9. Typical single-molecule traces for HJ4 S10
10. Linear range of calibration curves S11
11. Supporting references S11

Supplementary Table S1. A comparison of relevant multiplexing nucleic acid detection methods. Please note that other multiplexed techniques that achieved a lower LOD without target amplification and labeling have been demonstrated for 3 or fewer targets as compared to 4 targets using our HJ-based smFRET approach.

Multiplex Techniques	Target	Limit of detection (LOD)	Number of targets	Target Amplification	Target labeling
CRISPR	DNA, RNA	2 aM[1]	4	Yes	Yes
SMOS-qPCR	DNA, RNA	0.1 zM[2]	4	Yes	Yes
SERS with AuNPs	RNA	10 fM[3]	3	No	No
Electrochemical biosensors	RNA	0.25 -3.58 fM[4]	3	No	No
Quantum Dots	RNA	1.5 pM[5]	2	Yes	No
Double-enhanced SERS nanosensors	DNA	0.839 fM[6]	3	No	No
Hybrid-multitrap optical tweezers	RNA	0.27-0.33 fM[7]	3	No	No
(MoS_2-DMA) FRET	DNA	50 pM[8]	5	No	Yes
Electro-optical sensing platform	RNA	5-8 fM[9]	2	No	No
Fluorescent biosensor	DNA	20 pM[10]	3	Yes	No
Encoded hydrogel microparticle assay	miRNA	2.4 aM[11]	3	No	Yes
Microgel-based fluorescence assay	miRNA	2.6 fM[12]	3	No	Yes
HJ-based smFRET (Our method)	DNA	10-50 fM	4	No	No

CRISPR: Clustered Regularly Interspaced Short Palindromic Repeats; SMOS-qPCR: Sensitive and Multiplexed One-Step-quantitative Polymerase Chain Reaction; SERS: Surface-enhanced Raman Spectroscopy; AuNPs: Gold Nanoparticles; MoS_2-DMA: Molybdenum disulfide–Droplet Microarray; FRET: Fluorescence Resonance Energy Transfer; HJ: Holliday Junction; smFRET: single-molecule FRET.

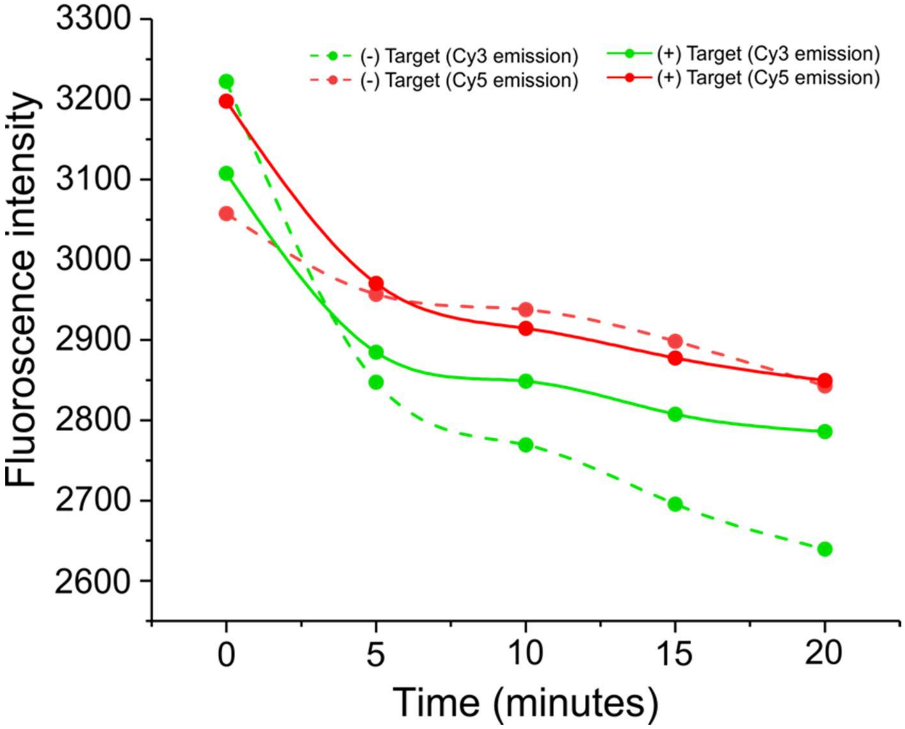

Supplementary Figure S1. Multiplex bulk FRET of four selected 4-way junctions. In bulk FRET experiments, only Cy3 was excited and the fluorescence intensities of both the Cy3 and Cy5 fluorophores were recorded in the absence and in the presence of target over time. Since bulk FRET measures the average of all the molecules, four HJs were not distinguishable in the mixture. All the experiments were carried out with 30 nM sensor. The target concentration was 1 μM.

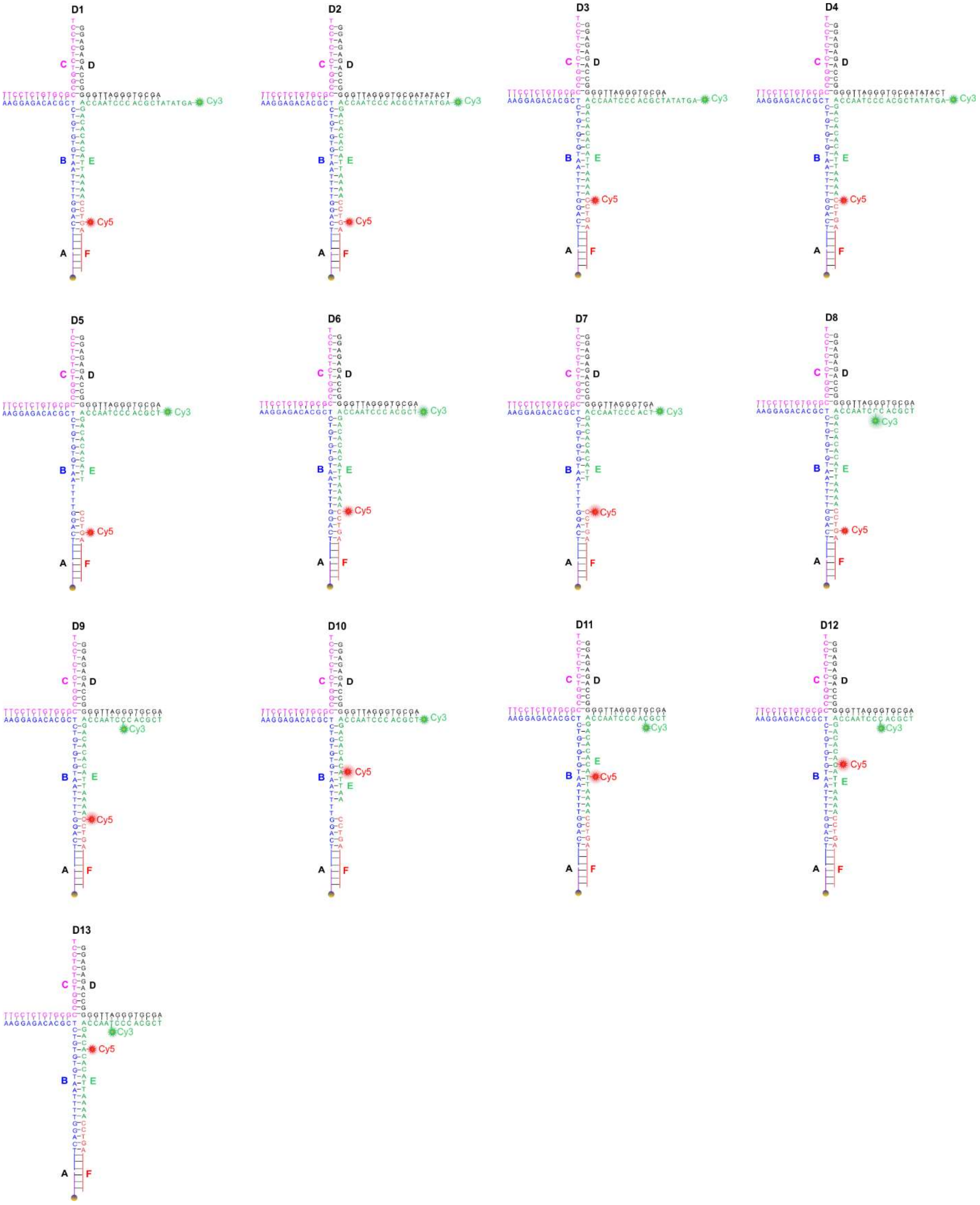

Supplementary Figure S2. Designs of 4-way junctions. Thirteen different fluorophore labeling schemes (D1-D13) were custom designed by positioning the Cy3 and Cy5 fluorophores at different positions to determine the ones yielding non-overlapping FRET efficiencies. The target strand used was the same for all the designs for this preliminary screening.

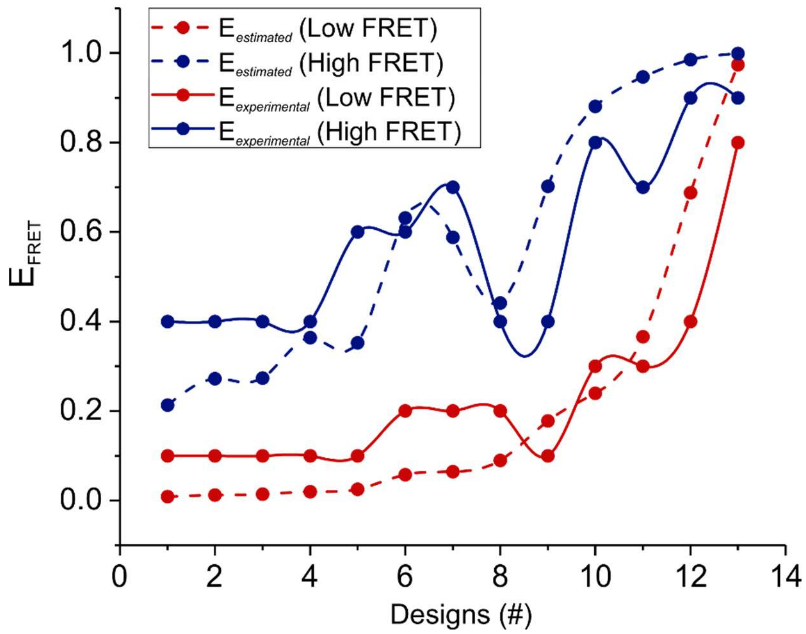

Supplementary Figure S3. Estimated (dashed) and experimental (solid) FRET efficiencies of the high- and low-FRET conformations of the HJs for 13 different labeling schemes. The inter-dye distance (R) and the estimated FRET efficiencies ($E_{\text{estimated}}$) were calculated using the equations below.

$$a^2 = b^2 + c^2 - 2bc \ \cos(A) \qquad \textbf{(Eq. 1)}$$

$$E = \frac{1}{1 + (R/R_0)^6} \qquad \textbf{(Eq. 2)}$$

Where a is the inter-dye distance (R), A is the inter-helix angle (the reported inter-helix angle for iso-I is 59° and iso-II is 121°)[13–16], R_0 is inter-dye distance at 50% FRET efficiency (5.4 nm for Cy3/Cy5 pair[17]). The length of b and c were calculated using 0.34 nm height per base pair for the dsDNA and 0.45 per nucleotide in the ssDNA. Possible folding of single nucleotide sequences, dye linkers and local environment impute the slight discrepancy in estimated and experimental FRET efficiencies.

Supplementary Table S2. List of DNA sequences used to prepare the four selected HJs (designs D8, D6, D11, and D12 from Figure S2). The target binding region is shown in red. Please note that the Strand F3 is same for HJ3 and HJ4.

Design (D)	**Strand name**	**Sequence**
**D8-> HJ1**	Strand A(Biotin)	/5BiotinTEG/ACG CGC TGG GCT ACG TCT TGC TGG CCG CAT
	Strand B-107	CTG TGC GGT ATT TCA CAC CGT TAG CTC AGG TTT TAA TGT GTG TCT TAC AAT GCT GCT
	Strand D-107	TGA TAG CCC TGG GTT AGG GTG CGA
	Strand E1	TCG CAC C/iCy3/CT AAC CAG ACA CAC ATT AAA A
	Strand F1	CCT G/iCy5/AG CTA ACG GTG TGA AAT ACC GCA CAG ATG CGG CCA GCA AGA CGT AGC CCA GCG CGT
**D6 -> HJ2**	Strand A(Biotin)	/5BiotinTEG/ACG CGC TGG GCT ACG TCT TGC TGG CCG CAT
	Strand B-342-3p	CTG TGC GGT ATT TCA CAC CGT TAG CTC AGG TTT TAA TGT GTG TCT TCT GTG TGA GA
	Strand-D-342-3p	ACG GGT GCG ATT GGT TAG GGT GCG A
	Strand E2	/5Cy3/TCG CAC CCT AAC CAG ACA CAC ATT AAA A
	Strand F2	/5Cy5/CCT GAG CTA ACG GTG TGA AAT ACC GCA CAG ATG CGG CCA GCA AGA CGT AGC CCA GCG CGT
**D11-> HJ3**	Strand A(Biotin)	/5BiotinTEG/ACG CGC TGG GCT ACG TCT TGC TGG CCG CAT
	Strand B-18b-5p	CTG TGC GGT ATT TCA CAC CGT TAG CTC AGG TTT TAA TGT GTG TCT GAT GCA CCT TA
	Strand-D-18b-5p	CTA ACT GCA CTA GGT TAG GGT GCG A
	Strand E3	TCG C/iCy3/AC CCT AAC CAG ACA CAC AT/iCy5/T AAA A
	Strand F3	CCT GAG CTA ACG GTG TGA AAT ACC GCA CAG ATG CGG CCA GCA AGA CGT AGC CCA GCG CGT
**D12-> HJ4**	Strand A(Biotin)	/5BiotinTEG/ACG CGC TGG GCT ACG TCT TGC TGG CCG CAT
	Strand B-92a-3p	CTG TGC GGT ATT TCA CAC CGT TAG CTC AGG TTT TAA TGT GTG TCT CAA GTG CAA TA
	Strand-D-92a-3p	ACA GGC CGG GAG GTT AGG GTG CGA
	Strand E4	TCG CAC/iCy3/ CCT AAC CAG ACA CAC/iCy5/ ATT AAA A
	Strand F3	CCT GAG CTA ACG GTG TGA AAT ACC GCA CAG ATG CGG CCA GCA AGA CGT AGC CCA GCG CGT

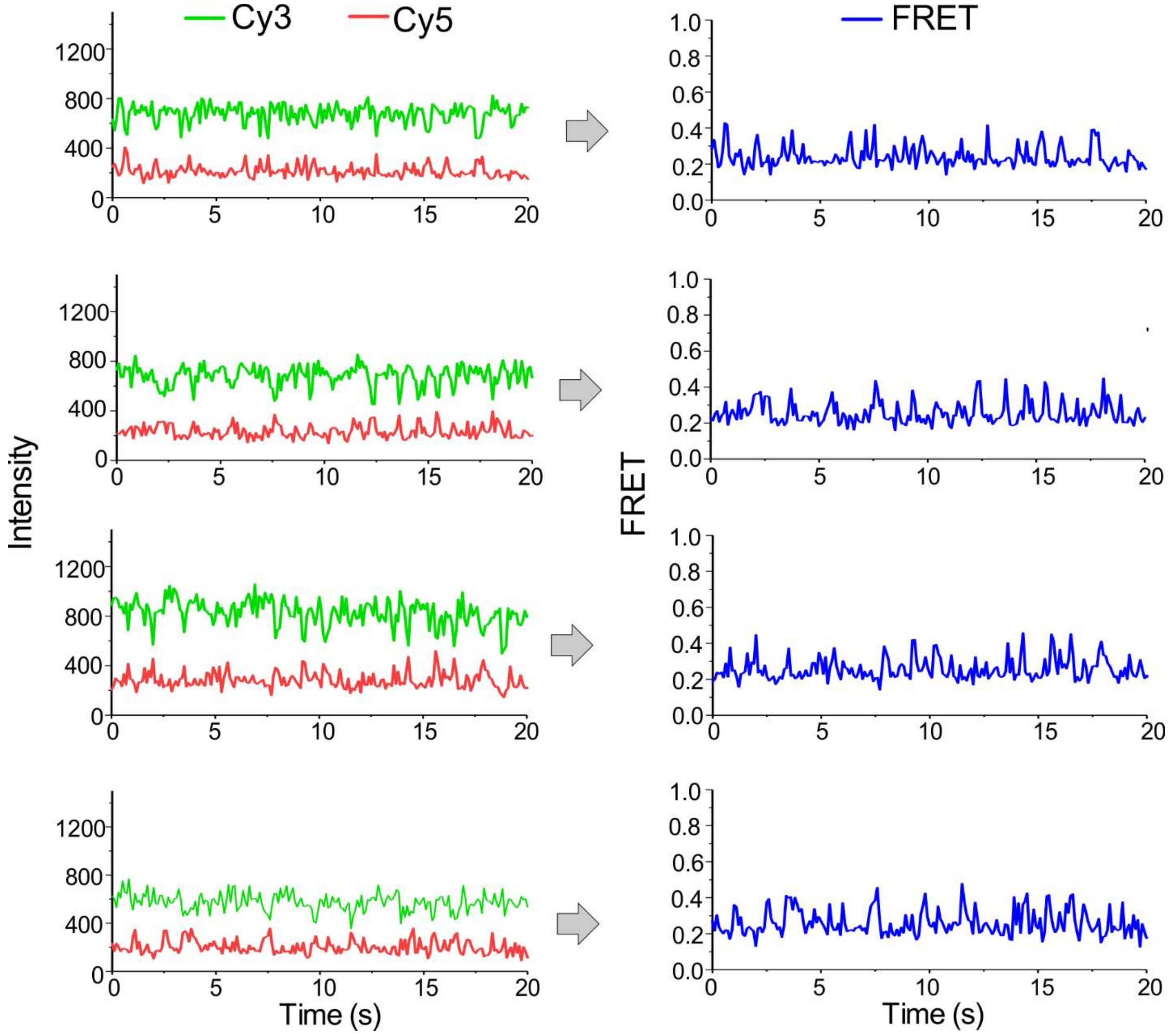

Supplementary Figure S4. Typical single-molecule traces from the single-plex detection of DNA mimic of miR-107 using HJ1. Intensity-time traces are shown on the left and the corresponding FRET-time traces are shown on the right. The molecules showed a clear anticorrelation between the Cy3 and Cy5 intensity with a low FRET of ~0.2 and a high FRET of ~0.4. Experiment was performed at 23°C (room temperature).

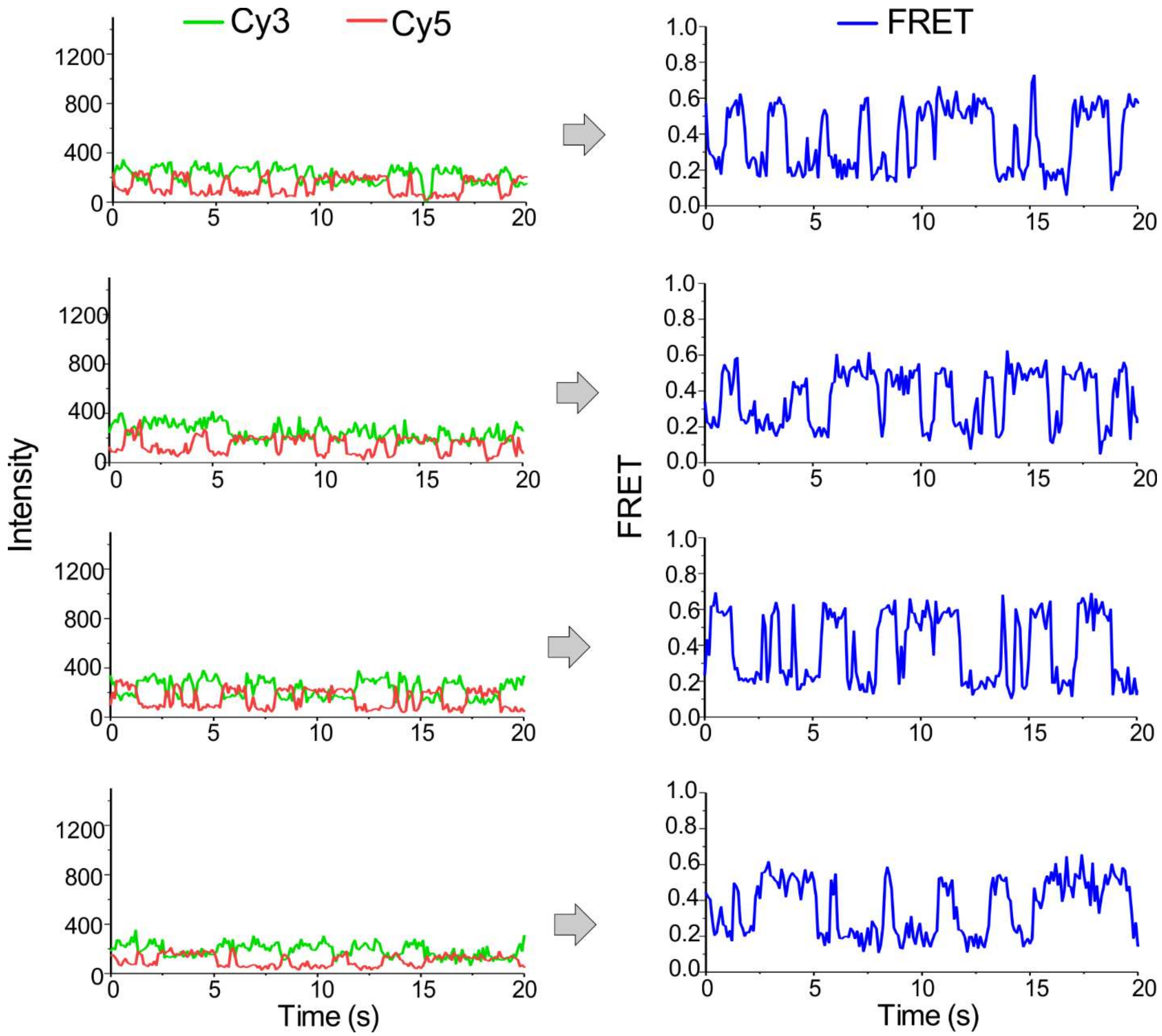

Supplementary Figure S5. Typical single-molecule traces from the single-plex detection of DNA mimic of miR-342-3p using HJ2. Intensity-time traces are shown on the left and the corresponding FRET-time traces are shown on the right. The molecules showed a clear anticorrelation between the Cy3 and Cy5 intensity with a low FRET of ~0.2 and a high FRET of ~0.6. Experiment was performed at 23°C (room temperature).

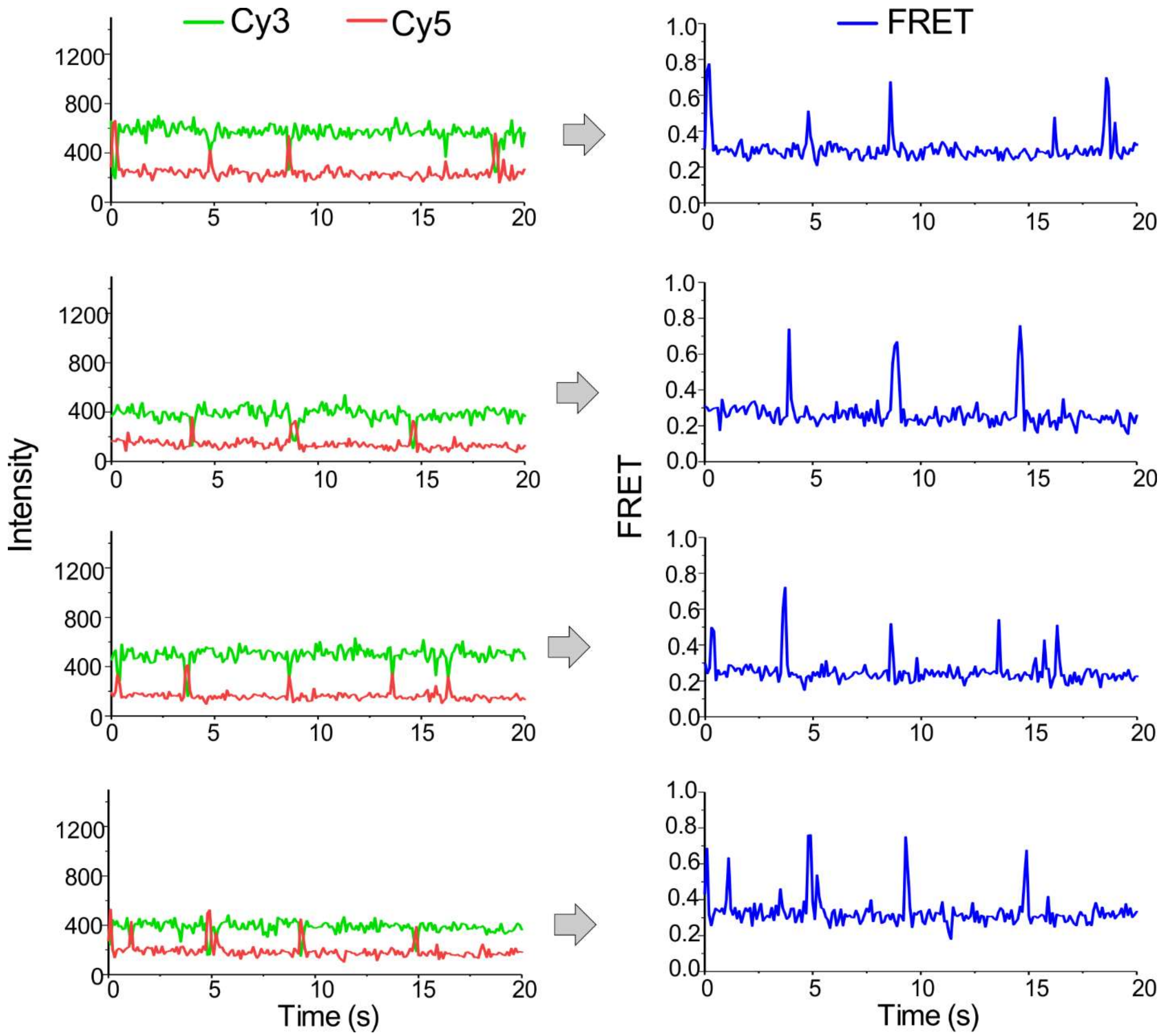

Supplementary Figure S6. Typical single-molecule traces from the single-plex detection of DNA mimic of miR-18b-3p using HJ3. Intensity-time traces are shown on the left and the corresponding FRET-time traces are shown on the right. The molecules showed a clear anticorrelation between the Cy3 and Cy5 intensity with a low FRET of ~0.3 and a high FRET of ~0.7. Experiment was performed at 23°C (room temperature).

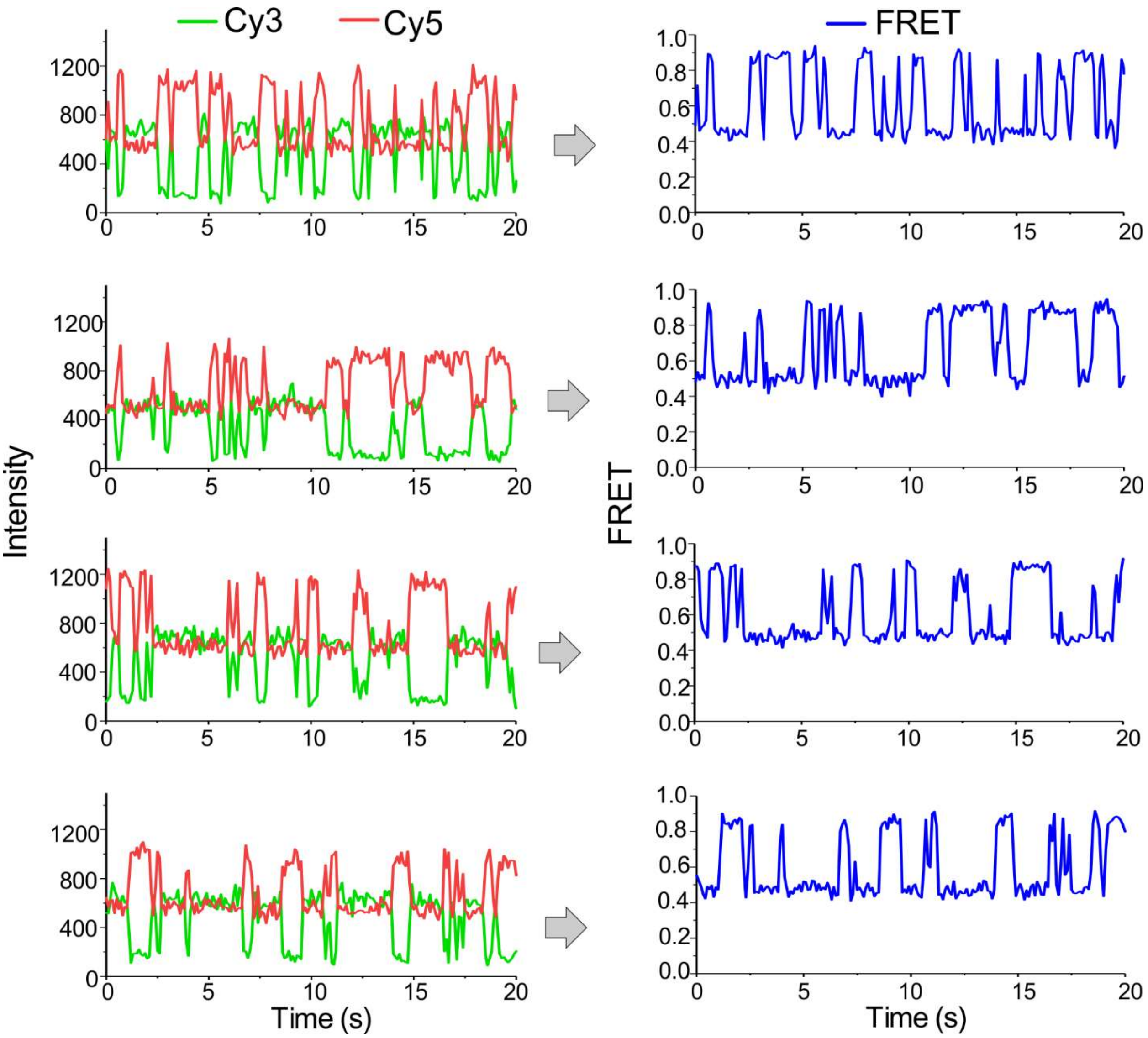

Supplementary Figure S7. Typical single-molecule traces from the single-plex detection of DNA mimic of miR-92a-3p using HJ4. Intensity-time traces are shown on the left and the corresponding FRET-time traces are shown on the right. The molecules showed a clear anticorrelation between the Cy3 and Cy5 intensity with a low FRET of ~0.4 and a high FRET of ~0.9. Experiment was performed at 23°C (room temperature).

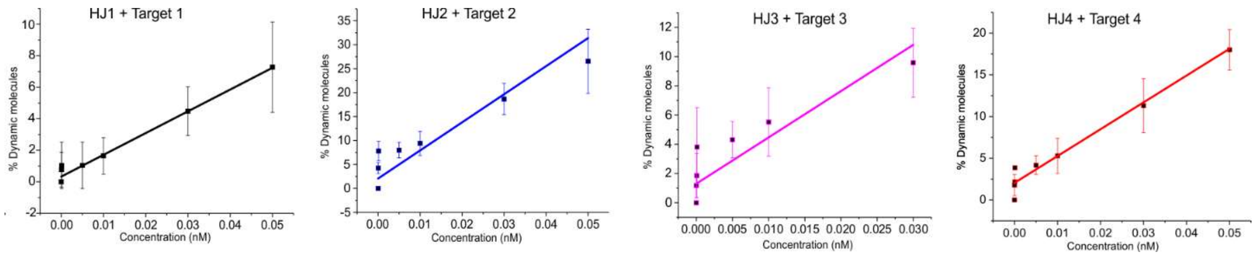

Supplementary Figure S8. Determination of concentration dependency. Each plot represents a linear range of the calibration curve with %Dynamic molecules as a function of target concentration. Target used (Target 1-4) were DNA mimics of miR-107, miR-342-3p, miR-18b-5p, and miR-92a-3p respectively. All the experiments were carried out at room temperature and about 100 single molecules were selected at each concentration to determine the percentage of dynamic molecules.

References

(1) Gootenberg, J. S.; Abudayyeh, O. O.; Kellner, M. J.; Joung, J.; Collins, J. J.; Zhang, F. Multiplexed and Portable Nucleic Acid Detection Platform with Cas13, Cas12a, and Csm6. *Science* **2018**, *360* (6387), 439–444. https://doi.org/10.1126/science.aaq0179.

(2) Xue, Y.; Wang, K.; Jiang, Y.; Dai, Y.; Liu, X.; Pei, B.; Li, H.; Xu, H.; Zhao, G. An Ultrasensitive and Multiplexed miRNA One-Step Real Time RT-qPCR Detection System and Its Application in Esophageal Cancer Serum. *Biosens. Bioelectron.* **2024**, *247*, 115927. https://doi.org/10.1016/j.bios.2023.115927.

(3) Zhou, W.; Tian, Y.-F.; Yin, B.-C.; Ye, B.-C. Simultaneous Surface-Enhanced Raman Spectroscopy Detection of Multiplexed MicroRNA Biomarkers. *Anal. Chem.* **2017**, *89* (11), 6120–6128. https://doi.org/10.1021/acs.analchem.7b00902.

(4) Pimalai, D.; Putnin, T.; Waiwinya, W.; Chotsuwan, C.; Aroonyadet, N.; Japrung, D. Development of Electrochemical Biosensors for Simultaneous Multiplex Detection of microRNA for Breast Cancer Screening. *Microchim. Acta* **2021**, *188* (10), 329. https://doi.org/10.1007/s00604-021-04995-8.

(5) Jie, G.; Zhao, Y.; Wang, X.; Ding, C. Multiplexed Fluorescence Detection of microRNAs Based on Novel Distinguishable Quantum Dot Signal Probes by Cycle Amplification Strategy. *Sens. Actuators B Chem.* **2017**, *252*, 1026–1034. https://doi.org/10.1016/j.snb.2017.06.107.

(6) Guo, R.; Yin, F.; Sun, Y.; Mi, L.; Shi, L.; Tian, Z.; Li, T. Ultrasensitive Simultaneous Detection of Multiplex Disease-Related Nucleic Acids Using Double-Enhanced Surface-Enhanced Raman Scattering Nanosensors. *ACS Appl. Mater. Interfaces* **2018**, *10* (30), 25770–25778. https://doi.org/10.1021/acsami.8b06757.

(7) Yu, H.; Jia, Z.-S.; Xu, P.-F.; Liu, Y.; Xu, D.-D.; Li, Y.-Y.; Tang, H.-W. Multiple miRNA Detection through a Suspended Microbead Array Encoded by Triple-Color Upconversion Luminescent Nanotags via Bi-Beam Splitter Hybrid-Multitrap Optical Tweezers. *Anal. Chem.* **2023**, *95* (37), 14086–14093. https://doi.org/10.1021/acs.analchem.3c02842.

(8) Oudeng, G.; Benz, M.; Popova, A. A.; Zhang, Y.; Yi, C.; Levkin, P. A.; Yang, M. Droplet Microarray Based on Nanosensing Probe Patterns for Simultaneous Detection of Multiple

HIV Retroviral Nucleic Acids. *ACS Appl. Mater. Interfaces* **2020**, *12* (50), 55614–55623. https://doi.org/10.1021/acsami.0c16146.
(9) Cai, S.; Pataillot-Meakin, T.; Shibakawa, A.; Ren, R.; Bevan, C. L.; Ladame, S.; Ivanov, A. P.; Edel, J. B. Single-Molecule Amplification-Free Multiplexed Detection of Circulating microRNA Cancer Biomarkers from Serum. *Nat. Commun.* **2021**, *12* (1). https://doi.org/10.1038/s41467-021-23497-y.
(10) Hu, R.; Liu, T.; Zhang, X.-B.; Huan, S.-Y.; Wu, C.; Fu, T.; Tan, W. Multicolor Fluorescent Biosensor for Multiplexed Detection of DNA. *Anal. Chem.* **2014**, *86* (10), 5009–5016. https://doi.org/10.1021/ac500618v.
(11) Lee, H.; Shapiro, S. J.; Chapin, S. C.; Doyle, P. S. Encoded Hydrogel Microparticles for Sensitive and Multiplex microRNA Detection Directly from Raw Cell Lysates. *Anal. Chem.* **2016**, *88* (6), 3075–3081. https://doi.org/10.1021/acs.analchem.5b03902.
(12) Causa, F.; Aliberti, A.; Cusano, A. M.; Battista, E.; Netti, P. A. Supramolecular Spectrally Encoded Microgels with Double Strand Probes for Absolute and Direct miRNA Fluorescence Detection at High Sensitivity. *J. Am. Chem. Soc.* **2015**, *137* (5), 1758–1761. https://doi.org/10.1021/ja511644b.
(13) Hargreaves, D.; Rice, D. W.; Sedelnikova, S. E.; Artymiuk, P. J.; Lloyd, R. G.; Rafferty, J. B. Crystal Structure of E.Coli RuvA with Bound DNA Holliday Junction at 6 Å Resolution. *Nat. Struct. Biol.* **1998**, *5* (6), 441–446. https://doi.org/10.1038/nsb0698-441.
(14) Gibbs, D. R.; Dhakal, S. Single-Molecule Imaging Reveals Conformational Manipulation of Holliday Junction DNA by the Junction Processing Protein RuvA. *Biochemistry* **2018**, *57* (26), 3616–3624. https://doi.org/10.1021/acs.biochem.8b00404.
(15) Megalathan, A.; Wijesinghe, K. M.; Dhakal, S. Single-Molecule FRET-Based Dynamic DNA Sensor. *ACS Sens.* **2021**, *6* (3), 1367–1374. https://doi.org/10.1021/acssensors.1c00002.
(16) Watson, J. Definitions and Analysis of DNA Holliday Junction Geometry. *Nucleic Acids Res.* **2004**, *32* (10), 3017–3027. https://doi.org/10.1093/nar/gkh631.
(17) Dhakal, S.; Adendorff, M. R.; Liu, M.; Yan, H.; Bathe, M.; Walter, N. G. Rational Design of DNA-Actuated Enzyme Nanoreactors Guided by Single Molecule Analysis. *Nanoscale* **2016**, *8* (5), 3125–3137. https://doi.org/10.1039/C5NR07263H.
